# Treatment and control of bovine hypodermosis with ivermectin long-acting injection (IVOMEC® GOLD)

**DOI:** 10.1186/s13071-016-1823-8

**Published:** 2016-10-18

**Authors:** Domenico Otranto, Greg Johnson, Kevin Syvrud, Stephen Yoon, James S. Hunter, Steffen Rehbein

**Affiliations:** 1 Department of Veterinary Medicine, University of Bari, Str. prov. per Casamassima km 3, 70010 Valenzano (Bari), Italy; 2 Department of Animal and Range Sciences, Montana State University, Bozeman, MT USA; 3 Summit Research, Helena, MT USA; 4 Merial, Inc., 3239 Satellite Blvd., Duluth, GA 30096 USA; 5 Merial GmbH, Kathrinenhof Research Center, Walchenseestr. 8-12, 83101 Rohrdorf, Germany

**Keywords:** Ivermectin, Long-acting injection, Efficacy, *Hypoderma bovis*, *Hypoderma lineatum*, Cattle

## Abstract

**Background:**

The studies reported here were conducted to assess the efficacy of ivermectin long-acting injection (IVM LAI; IVOMEC® GOLD, Merial; 3.15 % w/v ivermectin) for the treatment and control of natural infestations of cattle by *Hypoderma bovis* and *Hypoderma lineatum*, which are the most economically important oestrid flies of cattle in the northern hemisphere.

**Methods:**

Cattle selected from herds with a history of *Hypoderma* infestation were grouped into blocks of three (Italy, 33 cattle; Germany, 30 cattle) or two (USA, 16 cattle) animals each, on the basis of positivity at the pre-treatment anti-*Hypoderma* antibody titres. Within each block, animals were randomly allocated to one of the following treatment regimens: saline (control); IVM LAI, administered at the predicted time of occurrence of first-instar larvae (Italy, Germany, USA); IVM LAI, administered at the predicted time of occurrence of second- and/or third-instar larvae (Italy, Germany). All treatments were administered by subcutaneous injection in correspondence of the area anterior to the shoulder at 1 ml/50 kg body weight, which corresponds to 630 mcg IVM/kg for IVM LAI.

**Results:**

No *Hypoderma* larvae emerged from animals treated with IVM LAI, whereas live *H. lineatum* (Italy) or *H. bovis* (Germany, USA) larvae were collected from saline-treated animals (*P* < 0.01). No adverse reactions to treatments were in any of the animals enrolled in the study.

**Conclusions:**

The results from this study demonstrate that ivermectin in a long-acting formulation is 100 % efficacious in the treatment of cattle naturally infested by *H. bovis* and *H. lineatum* larvae at all stages of development. IVM LAI can, therefore, be used as ‘prophylactic’ treatment for *Hypoderma* spp. infestations in absence of external evidence of their presence and thus prior to skin and carcass damage, and as ‘therapeutic’ treatment, when warbles are already present.

## Background

The family Oestridae includes some 150 species of dipteran flies whose larvae are obligate parasites living, over weeks to months, in the tissues or organs of domestic and wild animals and causing obligate myiases [1, 2]. The oestrid genus *Hypoderma* includes seven species of flies, of which three are known to cause bovine hypodermosis (also known as cattle grub or warble fly infestation) in the northern hemisphere, i.e. *Hypoderma bovis*, *H. lineatum* and *H. sinense*. During the summer season, female flies lay eggs; from these, first-instar larvae hatch to then migrate over several months through the somatic tissues of the animals via the oesophagus (*H. lineatum*, *H. sinense*) or the perirachidian tissues (*H. bovis*). Larvae moult into second- and third-instar larvae and form the typical subcutaneous nodules under the skin in the dorsal and lumbar regions of cattle (warbles) in late winter and spring [3, 4].

Though bovine hypodermosis does not induce significant mortality and morbidity, this infestation affects the productivity and welfare of animals, thus resulting in considerable losses to the livestock industry arising from a number of causes [2, 5, 6]. In addition, bovine *Hypoderma* species may accidentally infest humans and seldom cause severe conditions [7–9].

Administration of systemically active insecticides, i.e. topical formulations of organophosphates and products with macrocyclic lactone compounds, kill *Hypoderma* larvae during their migration through the animal’s body. Since the early 1980s, the widespread use of broad-spectrum parasiticides, particularly macrocyclic lactones, led to a marked decrease in the prevalence of bovine hypodermosis in many developed countries of the northern hemisphere [10]. This resulted in successful area-wide or national control programs. However, despite the extensive control measures undertaken, surveillance studies based on serum and milk serology, as well as reports on clinical infestation of cattle and parasiticide efficacy studies and/or description of human cases published over the past 15 years, provide evidence that residual populations of *Hypoderma* spp. are still present in cattle in North America including Canada and the USA, as well as in Europe, e.g. in Portugal, Spain, France, Italy, Belgium, Switzerland, Germany, Poland, Slovakia and Estonia [11–24]. In addition, bovine hypodermosis is prevalent in several countries in eastern and south eastern Europe including Belarus, Russia, Romania, Serbia, Bosnia and Herzegovina, Kosovo, Albania and Greece [25–33], and it is still endemic in resource-limited countries in northern Africa and Asia [34–41].

Macrocyclic lactones are characterized by excellent efficacy against a broad spectrum of nematode endo- and arthropod ectoparasites, including infestation with myiasis causing larvae of oestrid flies [42]. More recently, injectable long-action macrocyclic lactone formulations were authorized for use in cattle in several countries including ivermectin long-acting injection (IVM LAI; IVOMEC® GOLD, Merial) [43].

Earlier studies that reported IVM LAI is highly efficacious in the control of cattle infestations by larvae of the neotropical oestrid fly, *Dermatobia hominis* [44, 45]. The studies reported here were conducted to assess the efficacy of IVM LAI for the treatment and control of natural infestations of cattle by *H. bovis* and *H. lineatum*, the economically most important oestrid flies of cattle in the northern hemisphere.

## Methods

The studies were designed in accordance with and consistent to the World Association for the Advancement of Veterinary Parasitology guidelines for evaluating the efficacy of ectoparasiticides against myiasis causing parasites [46]. The studies were conducted in compliance with VICH GL9, entitled *Good Clinical Practice* and with the local legislation for animal welfare. The studies were performed as blinded studies, i.e. all personnel involved in collecting efficacy data were masked as to the assignment of each animal to treatment groups.

### Experimental animals

A total of 79 healthy beef cattle, weighing 121.5 to 386 kg at the start of the study (day -1 or day 0), and aging approximately 5–21 months, were included in three studies, conducted in Italy (Study 1), Germany (Study 2) and the USA (Study 3). Animal descriptions and details are presented in Table 1. The animals were sourced from herds with a history of *Hypoderma* spp. in the Basilicata region, Italy, Rhine Palatinate, Germany or Wisconsin, USA. Animals had not been previously treated with a macrocyclic lactone product and they were previously exposed to *Hypoderma* spp. as confirmed by the presence of anti-*Hypoderma* antibodies prior to treatment.

**Table 1:** Animal description and details

Study^a^	Treatment^b^, animals per treatment	Breed	Sex	~ Age (months)	Day -1/0 (kg)
1, Italy	Control (*n* = 11)	Podolica pugliese, Podoloca cross, Romagnola	7 male, 26 female	5–9	132–332
	IVM LAI L1^c^ (*n* = 11)				
	IVM LAI L2/L3^d^ (*n* = 11)				
2, Germany	Control (*n* = 10)	Beef crossbred	10 male, 20 female	6–21	121.5–386
	IVM LAI L1 (*n* = 10)				
	IVM LAI L2/L3 (*n* = 10)				
3, USA	Control (*n* = 8)	Angus cross, Hereford cross	8 male castrate, 8 female	7–8	205.5–373
	IVM LAI L1 (*n* = 8)				

^a^Source of animals: Study 1: Basilicata, Italy; Study 2: Rhine Palatinate, Germany; Study 3: Wisconsin, USA

^b^Control = Saline; IVM LAI = ivermectin long-acting injection (IVOMEC® GOLD)

^c^Treatment during the L1 stage of development

^d^Treatment during the L2/L3 stages of development

All animals were handled with due regard to their welfare and in compliance with Merial Institutional Animal Care and Use Committee (IACUC) approvals, any applicable local regulations, and requirements of any local IACUC.

### Experimental design

A randomized block design based on pre-treatment anti-*Hypoderma* antibody levels was used; each individual animal represented the experimental unit in each study. Eleven or ten blocks of three (Studies 1 and 2) or eight blocks of two (Study 3) cattle each were formed sequentially based on decreasing pre-treatment anti-*Hypoderma* antibody level results (Studies 1 and 2: Pourquier® ELISA Bovine Hypodermosis/Hypo Serums; Montpellier, France; Study 3: ELISA test as described by Colwell et al. [47]. Within each block, animals were randomly allocated to 1 of the following treatments: saline (control) (Studies 1, 2 and 3); IVM LAI (3.15 % w/v ivermectin in a LAI formulation; IVOMEC® GOLD, Merial), administered in correspondence of the predicted occurrence of first-instar larvae (Studies 1, 2 and 3); IVM LAI, administered in correspondence of the predicted occurrence of second- and/or third-instar larvae (Studies 1 and 2).

Cattle were either kept indoors and housed in loose-pens (Study 1) or individually stanchioned (Study 2) per block, or were kept as one group in a fenced dry lot (Study 3). Animals were fed as per local practice and were water was provided *ad libitum*.

The studies commenced either in November (Study 1) or in December (Studies 2 and 3). Treatments, saline as well as IVM LAI, were administered at 1 ml/50 kg bodyweight once in correspondence of the predicted occurrence of *Hypoderma* spp. first-instar larvae (day 0: all studies) or once in correspondence of the predicted occurrence of second- and third-instar larvae (day 84: Study 1; day 125: Study 2) by subcutaneous injection in an area anterior to the shoulder using commercial disposable syringes and needles. All cattle were observed hourly for 4 h post-treatment and thereafter once daily throughout the course of the studies for health problems or adverse drug reactions. Animals were weighed prior to treatments (days -1 and 84: Study 1; days 0 and 125: Study 2; day -1: Study 3) for dose calculation. Cattle were inspected for warbles starting 40 (Studies 1 and 2) or 28 (Study 3) days following day 0 treatment and then at intervals no greater than every 2 weeks until sufficient warbles were detected to warrant a second treatment in correspondence of the predicted occurrence of second- and third-instar larvae. Thereafter, cattle were inspected, and mature *Hypoderma* spp. larvae were collected, viability was determined and larvae were identified to species according to published keys [48, 49], unless identification was impossible because of decomposition prior to collection. Cattle were examined until the end of the studies, i.e. emergence of larvae could no longer be detected.

### Data analysis

*Hypoderma* live larval counts were transformed to the natural logarithm of (count + 1) for calculation of geometric means. IVM LAI treatment groups were compared (separately) to the saline-treated (control) group using the Wilcoxon rank sum test. A 2-sided test was used at the significance level of 0.05. Analyses were performed using SAS® Version 8.2. Efficacy was calculated as 100[(C-T)/C], where C is the geometric mean for the saline-treated (control) group and T is the geometric mean for the IVM LAI treated group.

## Results

All identifiable larvae collected in Study 1 were identified as *H. lineatum*, whereas all larvae collected in Studies 2 and 3 were identified as *H. bovis*.

Sufficient warbles to warrant treatment against second- and third-instar *Hypoderma* larvae were detected on study days 84 (Study 1: 3 to 49 warbles in 10/11 animals) and 125 (Study 2: 1 to 16 warbles in 8/10 animals), respectively. In correspondence of these days, 3 to 41 warbles were counted on 9/11 control animals in Study 1, and 1 to 14 warbles on 7/10 control animals in Study 2.

Following treatments against second- and third-instar larvae appearance, the number of warbles regressed to one warble each within 6 or 10 weeks in Study 2 and Study 1, respectively. At the inspection, the warbles contained a dead third-instar *Hypoderma* larva (Study 1) or granulomatous tissue (Study 2). No *Hypoderma* spp. larvae emerged from any of the cattle treated in correspondence to the predicted occurrence of first-instar larvae or when the *Hypoderma* spp. larvae were in the second and/or third stage.

Forty-nine live *H. lineatum* larvae and 23 and 66 live *H. bovis* larvae were collected from the saline-treated (control) animals in Studies 1, 2 and 3, respectively, emergence of larvae could no longer be observed (study days 158, 168, or 183 in Studies 1, 2 and 3, respectively).

Cattle treated with IVM LAI in correspondence to the predicted occurrence of *Hypoderma* spp. first or second and third-instar larvae had significantly fewer *H. lineatum* or *H. bovis* larvae emerging and/or expressed than the saline-treated (control) animals (Table 2). The efficacy of IVM LAI against larval stages of *H. lineatum* or *H. bovis* was 100 % (Table 2).

**Table 2:** Therapeutic efficacy of IVM LAI against natural infestations of first-stage and second- and third-stage larvae of *Hypoderma* spp

Study	Treatment^a^ (Study day)	NI/NG^b^	GM^c^ (Range)	Efficacy^d^ (%)	Z-value	*P*-value^e^
		Live *Hypoderma lineatum* larvae				
1. Italy	Control (day 0)	9/11	2.8 (0–17)	–		
	IVM LAI L1^f^ (day 0)	0/11	0	100	3.6150	0.0003
	IVM LAI L2/L3^g^ (day 84)	0/11	0	100	3.6150	0.0003
		Live *Hypoderma bovis* larvae				
2. Germany	Control (day 0)	7/10	1.5 (0–8)	–		–
	IVM LAI L1 (day 0)	0/10	0	100	3.0729	0.0021
	IVM LAI L2/L3 (day 125)	0/10	0	100	3.0729	0.0021
3. USA	Control (day 0)	6/8	1.8 (0–22)	–		–
	IVM LAI L1 (day 0)	0/8	0	100	2.8387	0.0045

^a^Control = Saline; IVM LAI = ivermectin long-acting injection (IVOMEC® GOLD)

^b^Prevalence: Number of cattle infested/Number of cattle in group

^c^Geometric mean live larval count [based on transformation to ln (count + 1)]

^d^Efficacy = 100× (GM Control – GM IVM LAI/GM Control)

^e^Probability from Wilcoxon rank sum test

^f^Treatment during the L1 stage of development

^g^Treatment during the L2/L3 stages of development

Animals were reported as normal during hourly observations for 4 h post-treatment, indicating that the treatment (either IVM LAI or saline) was well accepted. There were no drug related health problems or adverse drug events observed at any time during the studies.

## Discussion

The results presented herein demonstrate that ivermectin in a long-acting formulation is 100 % efficacious in the treatment of cattle naturally infested with *H. bovis* and *H. lineatum* larvae. IVM LAI can, therefore, be used as ‘prophylactic’ treatment for *Hypoderma* spp. infestations, i.e. in absence of external evidence of their presence and prior to carcass and skin damage, and as ‘therapeutic’ treatment, e.g. when warbles are already present. Similar results were reported previously by several authors who tested other commercial formulations containing ivermectin and other compounds of the macrocyclic lactone family (reviewed by [23, 42]).

Serodiagnosis of hypodermosis was used to select the study animals, as this is the only way to detect infested animals well before the appearance of the warbles; however, there is no correlation between titer levels and intensity of infestation [50–52]. Although anti-*Hypoderma* antibody positive animals were enrolled in the studies, warbles were only detected in 80 % of the saline-treated animals (Studies 1, 2 and 3; *n* = 29), as well as in the animals subjected to treatment in correspondence of the predicted occurrence of second- and/or third-instar larvae (Studies 1 and 2; *n* = 21), with infestation rates of 86.4, 75 and 75 % in Studies 1, 2 and 3, respectively. The 80 % overall infestation rate is consistent with observations from previous studies where the development of warbles was recorded in 81.4 % of the animals which had tested positive for anti-*Hypoderma* antibodies [23]. Similarly, numbers of warbles on the animals and numbers of *Hypoderma* larvae collected from the control animals in this study were similar to those reported in the same studies [23]. As discussed earlier, these findings are in line with the predictive value of the available ELISAs but may also reflect, at least in part, the high mortality of larvae in *Hypoderma* spp. infestations [23]. Although ELISA serology may over-estimate the percentage of cattle that develop warbles (clinical hypodermosis), it is considered to be a sensitive indication of exposure of cattle to grub infestation and thus a suitable measure for detection of low level, persistent bovine *Hypoderma* spp. populations [22].

## Conclusions

With respect to the efficacy profile of IVM LAI, which provides effective control of nematode infections in cattle for up three months [53, 54] and the high sensitivity of *Hypoderma* spp. to macrocyclic lactones in general [42] and ivermectin in particular [55, 56], it can be assumed that IVM LAI while the flies are at the adult developmental stage will also provide protection against cattle *Hypoderma* spp. larval infestation. *Hypoderma* spp. populations are known to (re)generate promptly if eradication or control measures are incomplete or if re-introduction occurs [57–60]. Thus, these parasites should not be ignored and further attention should be paid to cattle parasite management programs not only in regions where bovine hypodermosis is widespread and an important economic burden for the cattle industry, but also in regions where these parasites occur (currently) at very low prevalence with patchy distribution.

## Abbreviations

IACUC: Merial Institutional Animal Care and Use Committee
IVM LAI: Ivermectin long-acting injection
